# Community pharmacists’ attitudes toward practice-based research and their perceived utilization of scientific evidence

**DOI:** 10.1371/journal.pone.0264193

**Published:** 2022-03-15

**Authors:** Eman Alefishat, Anan S. Jarab, Suhaib Muflih, Abdel Wahab Aqeel

**Affiliations:** 1 Department of Pharmacology, College of Medicine and Health Science, Khalifa University of Science and Technology, Abu Dhabi, United Arab Emirates; 2 Department Biopharmaceutics and Clinical Pharmacy, Faculty of Pharmacy, The University of Jordan, Amman, Jordan; 3 Center for Biotechnology, Khalifa University of Science and Technology, Abu Dhabi, United Arab Emirates; 4 Department of Clinical Pharmacy, Faculty of Pharmacy, Jordan University of Science and Technology, Irbid, Jordan; Endeavour College of Natural Health, AUSTRALIA

## Abstract

**Background:**

Enhancing the contribution of practicing pharmacists into scientific evidence via practice-based research (PBR) is crucial in maintaining high-quality clinical practice and healthcare delivery. Involving community pharmacists in PBR can potentially can also help break barriers to the utilization of the current best evidence in everyday pharmacy practice. The impact of pharmacists’ attitude towards PBR on their utilization of current best evidence in pharmacy practice is understudied.

**Objectives:**

The aim of the study was to investigate the impact of community pharmacists’ attitudes toward PBR on their utilization of current best evidence, barriers for conducting PBR in clinical practice were also investigated.

**Methods:**

In this cross-sectional study, 169 community pharmacists working across Jordan filled a questionnaire to assess their attitudes towards PBR, barriers to PBR, and their utilization of the current best evidence in clinical practice.

**Results:**

Pharmacists in this study showed a positive attitude toward PBR (attitude mean score >3.5). A positive attitude towards PBR was associated with high utilization of the best current scientific evidence. We also investigated several barriers to PBR and their association with utilization those included; the lack of perceived benefits, lack of institutional support, and lack of self-engagements of community pharmacists to PBR. The lack of perceived benefit was found to be negatively associated with pharmacists’ utilization of the current best scientific evidence.

**Conclusion:**

In this study, pharmacists’ utilization of current best evidence was found to be significantly impacted by their attitude toward PBR. The current study findings highlight the importance of supporting, promoting, and facilitating PBR among community pharmacists which can potentially enhance their utilization of the current best evidence in their everyday pharmacy practice.

## Background

The use of evidence based medicine in pharmacy practice is essential to improve health-related outcomes. Evidence based medicine is defined as “the conscientious, explicit, and judicious use of current best evidence in making decisions about the care of individual patients” [1]. Current best evidence has been defined as “the best available external clinical evidence from clinically relevant research, often from basic sciences of medicine, but especially from patient centred clinical research” Current best evidence has been reported to overturn previously accepted measures in patient care such as diagnostic tests and treatments protocols and replaces them with the current safer and more efficacious options. The utilization of current best evidence in pharmacy practice, which is defined as the use of the best available evidence in clinical practice, is an essential component in the cycle of clinical interventions to improve the public’s health and help patients achieving their therapeutic goals [2, 3]. In order to undergo clinically reasonable interventions and make practice decisions, pharmacists need to utilize the current scientific evidence from the literature [4]. Although pharmacists’ utilization of current best evidence in their daily practice is still underachieved, it is increasing worldwide [3–5]. In Jordan, despite the positive pharmacists’ attitude towards utilizing the current best evidence in pharmacy practice, there are personal and institutional barriers that led to low adoption of evidence-based pharmacy practice [6]. Several factors have been described as barriers for utilizing the current best evidence in pharmacy practice; among those, high workload and lack of time are the most commonly reported barriers [4, 6, 7]. It was concluded that in order to increase evidence-based practice, more practice-based research (PBR) must be conducted [8]. PBR helped move the research into community practices and address critical clinical questions among large and diverse populations [9]. Networks have become a widely successful mechanism for organizing PBR within a community context.

Enhancing the contribution of practicing pharmacists into scientific evidence via PBR is crucial in maintaining high-quality clinical practice and healthcare delivery [10]. The engagement of community pharmacists with healthcare researchers in PBR was reportedly limited to those who are interested in research and believe in the importance of current best evidence and utilize it in their everyday practice [11–13]. Practice-based research networks (PBRNs) have been introduced to help build research skills and improve the quality of research. PBRNs have been shown to open channels of collaboration between healthcare providers and bridge the gap between academia and practicing pharmacists [14].

While several studies from Australia, Canada, and the UK showed that pharmacists’ involvement in PBR is increasing, pharmacists are still underrepresented, and their contributions to PBR have proved to be low [12, 15–17]. Data on the attitude towards PBR among pharmacists in the Middle East is scarce. One study from Saudi Arabia focusing on pharmacists with postgraduate degrees showed that despite pharmacists’ high interest in PBR, several barriers are hindering their actual involvement in research [18]. To our knowledge, PBR and the association between pharmacists’ attitude towards PBR and their utilization of current best evidence has not been investigated in Jordan.

PBR is essential for the future progression of pharmacy, as a profession, and for providing evidence of the benefits of proposed changes in practice to the patients’ health outcomes [19]. It can potentially play a role in increasing the adoption and utilization of the current best evidence among pharmacists. The aim of the present study was to investigate the association of community pharmacists’ utilization of current best evidence with their attitudes toward PBR and the barriers they face when conducting PBR during their clinical practice.

## Methods

### Study design and subjects

A face-to-face survey was provided to a sample of 220 pharmacists from different geographical areas in Jordan using convenient sampling teachnique. The pharmacists were randomly selected from a list of pharmacists who are registered at the Jordanian Pharmaceutical Association. The participating pharmacists were “the pharmacist in charge” (i.e. the pharmacist responsible for the safe and effective running of the registered pharmacy). Pharmacy students and Pharmacist assistants were excluded from the study. During a visit to their worksite, pharmacists were provided with a description of the study and its objectives by the research assistant, and those who agreed to participate (169 pharmacists) signed a consent form. The pharmacists were recruited over a period of four months from January through October, 2019.

The study was approved by the Institutional Review Board committee at the Jordan University of Science and Technology (Approval No. 22/132/2020).

### Questionnaire development

Based on the published literature, domains were selected to assess attitudes towards PBR and barriers to PBR among pharmacists in a community pharmacy setting [20, 21]. The final survey comprised five multiple-choice questions about sociodemographic variables, seven Likert scale questions about attitude toward PBR, six Likert scale questions about utilization of current best evidence, and 36 statements designed to assess PBR-related barriers. Participants’ responses on the 5-point Likert scales were combined to generate the respondent’s overall scores of utilization of current best evidence, attitude toward PBR, and barriers to PBR.

In addition to the literature, we used the help of seven experts in the field of clinical pharmacy and public health, with a minimum of ten years of experience. Those experts reviewed each of the domains separately; their feedback was used to amend the questionnaire to establish both face and content validity. The experts contributed to develop a well-defined and relevant statements to the constructs being measured. As a result, two items were deleted of the original draft before disseminating the survey, rewording through all studied domains was also performed based on their feedback which resulted in more concise and clear statements.

Data were collected from 20 pharmacists was used as a pilot study before proceeding to the final version of the questionnaire. The feedback from the pharmacists was very thorough, it gave us insights on needed rephrasing of five questions, as well as change in the sequence of two questions. The data from the 20 pharmacists who participated in the pilot study was not included in the main study.

The seven pharmacist’s attitude questions covered their beliefs of the value of PBR, their willingness, abilities, and engagement in PBR, and the six utilization questions covered their perceived utilization of current best evidence in clinical practice in the form of a 5-point Likert scale. Each item of the questionnaire was given from 1 representing strongly disagree to 5 representing strongly agree. Frequency distribution has been illustrated for the Likert scale items. The following ranking was followed: (1.00–2.33) Low score (Third Rank), (2.34–3.67) Medium score (Second Rank), (3.68–5) High score (First Rank). Cronbach’s alpha was used to examine the reliability of the attitude and utility, which was 0.79 and 0.77, respectively.

### Data analysis

Data were analyzed using the statistical package for the social sciences (SPSS) version 25. Descriptive statistics were used to describe the demographic characteristics, attitudes towards and barriers for PBR, and the utilization of current best evidence. A five-point Likert scale ranging from “strongly disagree” to “strongly agree” was used to evaluate the response of participants to the items in each domain. The student’s t-test (t-test) and one-way analysis of variance (ANOVA) are statistical procedures for comparing mean values between groups. The t-test is used to compare the means of predictor variables with two groups, whereas the ANOVA is used to compare the means of predictor variables with three or more groups. To predict the relationship between the dependent variables of interest (i.e., attitude and utilization) and other independent variables, linear regression models were applied. A *P* value of 0.05 implies that the findings are statistically significant.

Because the initial survey developed comprised 36 variables designed to assess PBR-related barriers, which could result in a large set of data, exploratory factor analysis (EFA) was performed. EFA with Varimax rotation was used to reduce the dimensionality of large data sets and avoid potential multicollinearity by identifying a smaller number of uncorrelated variables known as factors, which are required to represent the data [22]. Keiser-Meyer-Olkin’s (KMO) coefficient was 0.771, and Bartlett’s test of sphericity was significant at 0.003 level. The R-matrix had a determinant of 0.028, which was greater than 0.000001. These findings established that the data set was suitable for factor analysis. After removing items that loaded on multiple factors, the final model included 17 items that loaded on a three-factor solution model, namely A. Lack of perceived benefit of PBR, B. Lack of institutional support, and C. Lack of self participation in PBR. Cronbach’s coefficient The corresponding Cronbach’s alpha values were 0.79, 0.76, and 0.71. S2 Table provides further clarification.

## Results

In total, 169 community pharmacists agreed to participate in the study and successfully submitted the filled questionnaire. Around 30% of participating pharmacists were working in chain pharmacies. The majority of the respondents were female (64.5%) and in the “18–25” age group (46.7%). More than half of the participants attained a postgraduate degree in the field of Pharmacy (51.5%) and had less than five years of experience (59.8%). The participants’ characteristics are shown in Table 1.

**Table 1:** Frequency distribution of sociodemographic characteristics of participants (n = 169).

Variable	Frequency (%)
Gender	
Male	60 (35.5)
Female	109 (64.5)
Age group	
18–25	79 (46.7)
26–30	38 (22.5)
Above 30	52 (30.8)
Experience (years)	
Less than five years	101 (59.8)
Greater than or equal to five years	68 (40.2)
Levels of education/degree	
Pharmacist	24 (14.2)
Doctor of pharmacy	58 (34.3)
Pharmacists with a postgraduate degree (master or PhD)	87 (51.5)
Work setting	
Independent pharmacy	119 (70.4)
Chain pharmacy	50 (29.6)

**Fig 1:**
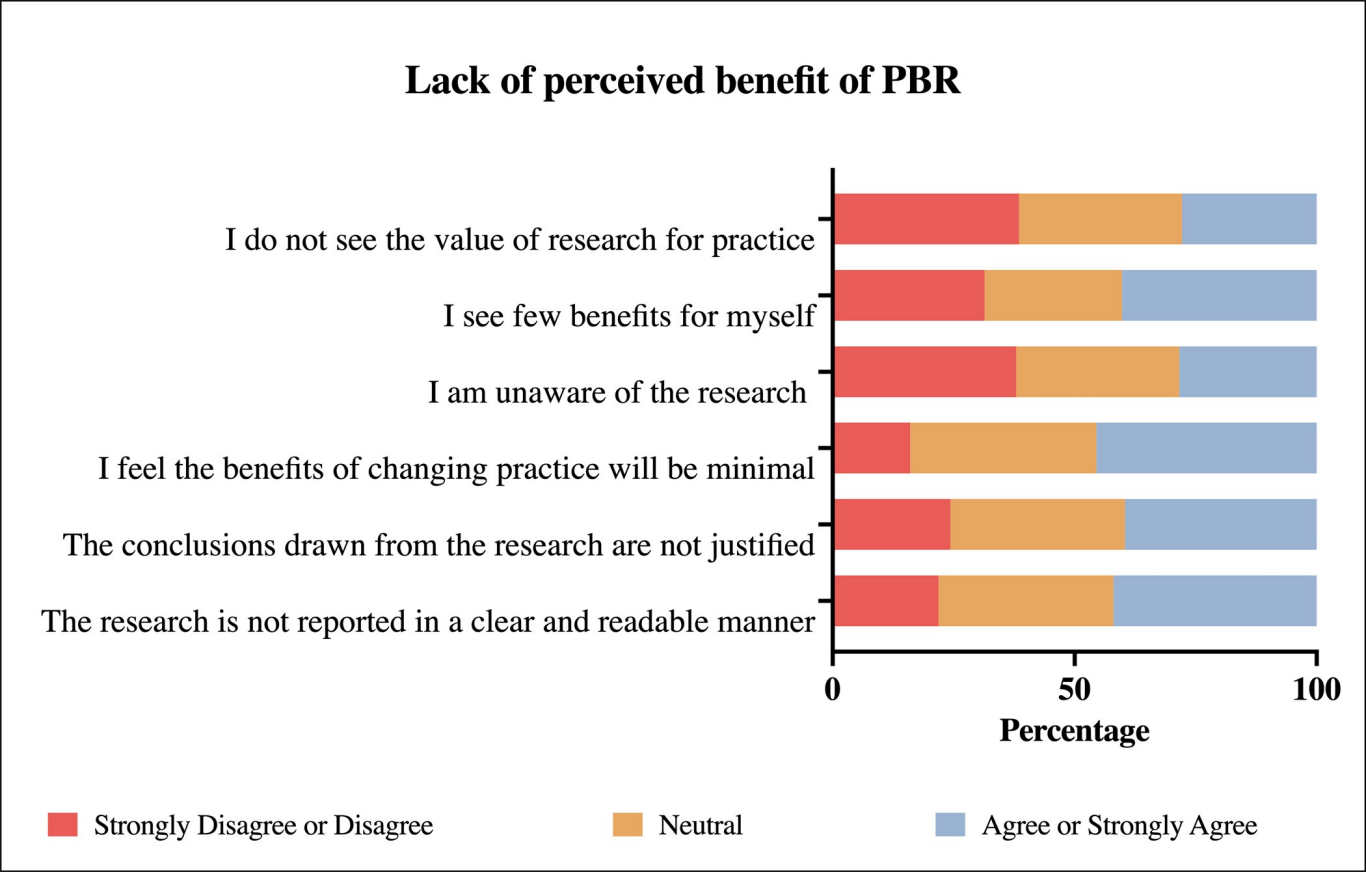
Item analysis of barrier A: Lack of perceived benefit of practice-based research (PBR).

**Fig 2:**
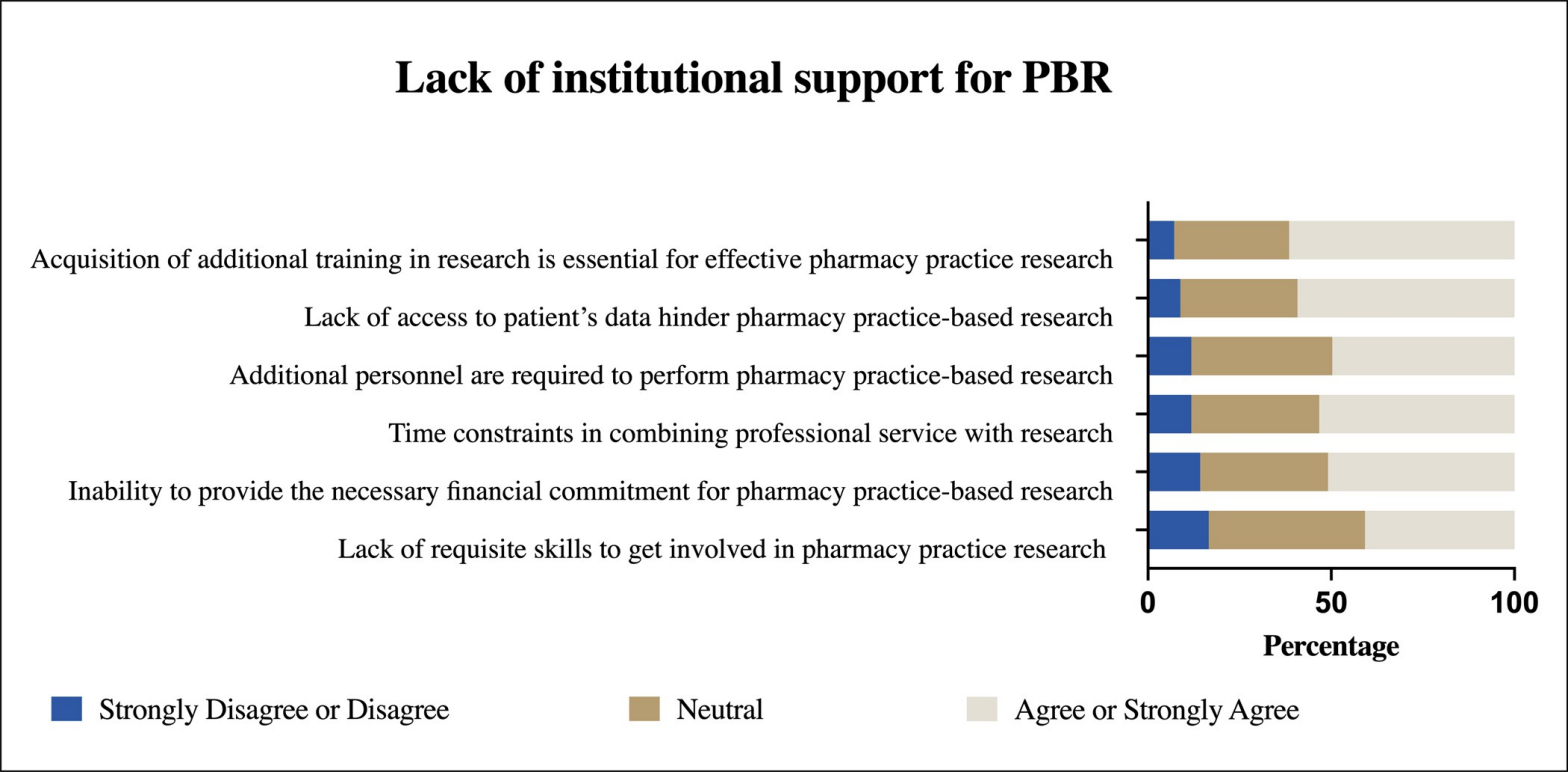
Item analysis of barrier B: Lack of institutional support for (PBR).

**Fig 3:**
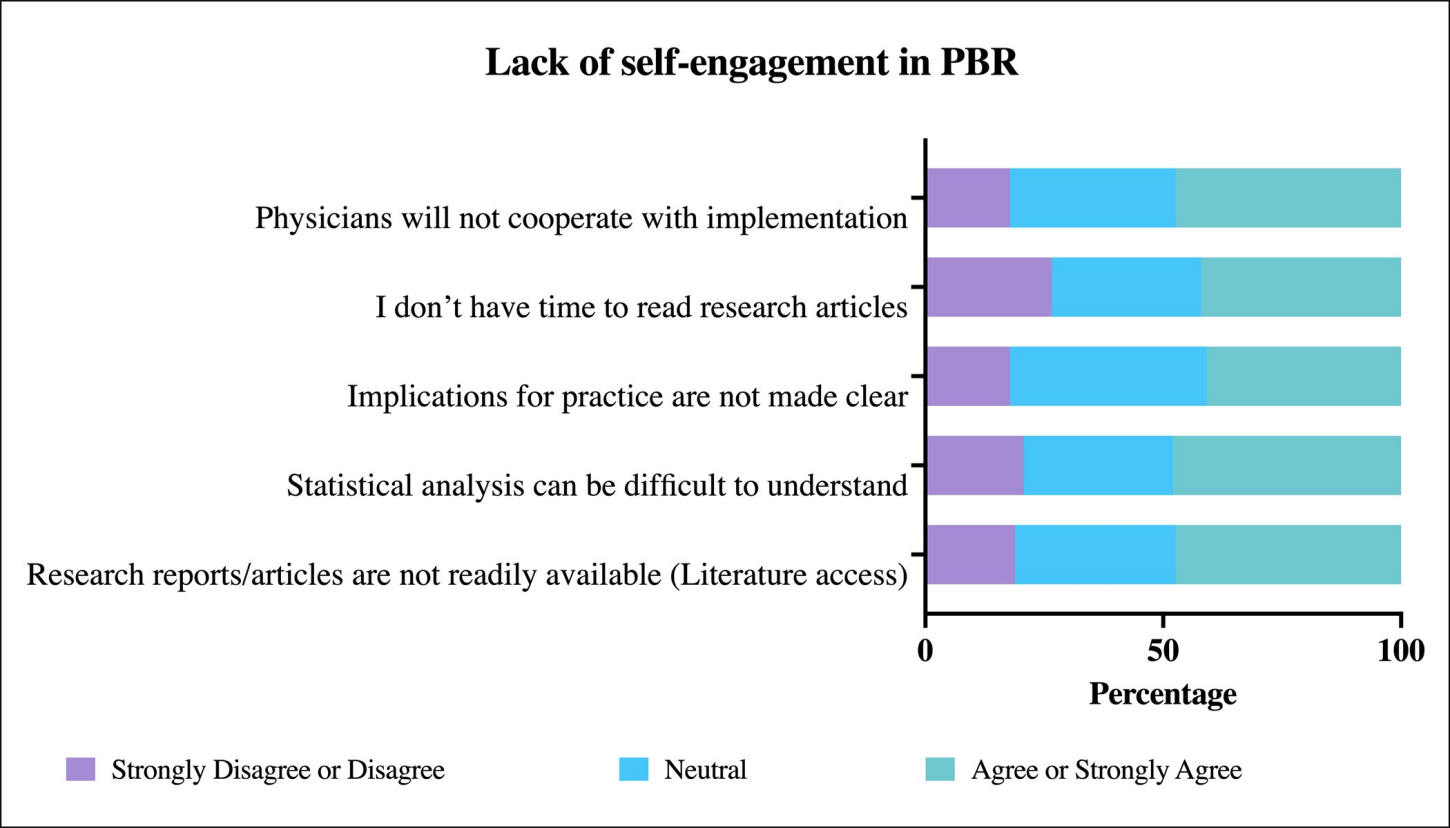
Item analysis of barrier C: Lack of self-engagement in (PBR).

The majority of participants expressed positive attitudes towards PBR and the use of clinical research tools in daily practice and were very receptive to the practical implementation of these research tools (Table 2). Pharmacists in this study showed high utilization of the current best evidence (mean scores >3.5) Table 3. However, participants reported several barriers to conducting PBR in their practice. Three groups of barriers have been identified: A: Lack of perceived benefit of PBR by community pharmacist (Fig 1), B: lack of institutional support (Fig 2), and C: lack of self-engagements in PBR (Fig 3).

**Table 2:** Descriptive statistics of pharmacists attitudes toward practice-based research (n = 169).

Statements	Strongly Disagree	Disagree	Neutral	Agree	Strongly Agree	Mean	Stand. Dev	Rank
	n (%)							
I like to read research studies related to pharmacy practice	1(0.6)	7(4.1)	43(25.4)	90(53.3)	28(16.6)	3.81	0.758	High
I shall be glad to be a part of research projects related to pharmacy practice	1(0.6)	15(8.9)	40(23.7)	76(45)	37(21.9)	3.79	0.895	High
I have faith in my capabilities to comprehend research and related terminologies concerned with pharmacy practice	3(1.8)	13(7.7)	61(36.1)	65(38.5)	27(16)	3.59	0.852	Medium
I am confident about my skills for designing research project related to pharmacy practice	2(1.2)	12(7.1)	63(37.3)	65(38.5)	27(16)	3.61	0.880	Medium
I am self-reliant in my skill for evaluating research terms of their application to pharmacy practice	0(0)	18(10.7)	62(36.7)	67(39.6)	22(13)	3.55	0.839	Medium
Pharmacy practice research is significant in recognizing and examining complications in pharmacy	0(0)	10(5.9)	52(30.8)	81(47.9)	26(15.4)	3.73	0.787	High
Pharmacy practice research is vital in pharmacy decision-making	0(0)	8(4.7)	46(27.2)	78(46.2)	37(21.9)	3.85	0.789	High
The overall Attitude score						3.7	0.83	High

**Table 3:** Descriptive statistics of pharmacists responses pertaining to research utilization in pharmacy practice (n = 169).

Statements	Strongly Disagree	Disagree	Neutral	Agree	Strongly Agree	Mean	Stand. Dev.	Rank
	Frequency (%)							
Using research in daily practice	3(1.8)	17(10.1)	62(36.7)	53(31.4)	34(20.1)	3.58	0.979	Medium
Using databases or search engines to search journal articles	2(1.2)	10(5.9)	43(25.4)	79(46.7)	35(20.7)	3.8	0.877	High
Ability to identify clinical problems by using research and journal clubs	0 (0)	10(5.9)	46(27.2)	91(53.8)	22(13)	3.74	0.758	High
Establish current best practices	3(1.8)	10(5.9)	59(34.9)	70(41.4)	27(16)	3.64	0.883	Medium
Being up to date with research to improve drug selection	0 (0)	14(8.3)	42(24.9)	79(46.7)	34(20.1)	3.79	0.86	High
Reviewing research to help pharmacist with drug monitoring in daily practice	0 (0)	8(4.7)	53(31.4)	58(34.3)	50(29.6)	3.89	0.889	High
The overall Utilization score						3.74	0.87	High

There were no significant differences in pharmacists’ overall attitudes regarding PBR and utilization of best current scientific evidence in pharmacy practice based on their age or gender. There was a statistically significant variation in pharmacists’ overall attitudes toward PBR based on their educational levels and workplace settings. Responses to the utilization of current best evidence in pharmacy practice varied significantly according toyears of experiencewith more experienced pharmacists having a lower utilization score. The results are illustrated in Table 4.

**Table 4:** T-test and F-test for pharmacists’ attitudes toward practice-based research and their utilization of current best evidence by sociodemographic characteristics.

Variables		Attitudes		Utilization of current best evidence	
	N = 169	Mean (SD)	*p*-value	Mean (SD)	*p*-value
Gender					
Male	60	3.7 (0.34)	0.740	3.73 (0.51)	0.813
Female	109	3.69 (0.41)		3.74 (0.53)	
Age group					
18–25	79	3.67 (0.41)	0.611	3.78 (0.54)	0.466
26–30	38	3.69 (0.42)		3.67 (0.48)	
Above 30	52	3.74 (0.44)		3.73 (0.51)	
Experience (years)					
Less than 5 years	101	3.68 (0.46)	0.342	3.82 (0.50)	0.016*
Greater than or equal to 5 years	68	3.71 (0.48)		3.61 (0.49)	
Levels of education/degree					
Pharmacist	24	3.52 (0.41)	0.004*	3.78 (0.54)	0.665
Doctor of pharmacy	58	3.88 (0.57)		3.77 (0.48)	
Pharmacists with a postgraduate degree (master or PhD)	87	3.60 (0.53)		3.69 (0.49)	
Work setting					
Independent pharmacy	119	3.78 (0.51)	0.045*	3.76 (0.53)	0.657
Chain pharmacy	50	3.56 (0.46)		3.71 (0.75)	

A multiple linear regression model was generated. The forward and backward stepwise selection methods revealed that attitudes and lack of perceived benefit of PBR were significant variables that best fit the data (see Table 5, and S1 Table). The adjusted coefficient of multiple determination (R^2^) value was 0.39.

**Table 5:** Predictors of pharmacists’ utilization of current best evidence in their practice.

Parameter	Utilization score		
	B	Std. Error	*p*-value
Gender			
Male (*Reference*)	-	-	-
Female	0.056	0.077	0.472
Age group			
18–25 (*Reference*)	-	-	-
26–30	-0.093	0.089	0.297
Above 30	-0.176	0.104	0.083
Experience (years)			
Less than five years (*Reference*)	-	-	-
Equal to or greater than five years	-0.151	0.081	0.059
Levels of education/degree			
Pharmacist (*Reference*)	-	-	-
Doctor of pharmacy	0.122	0.121	0.315
Pharmacists with a postgraduate degree (master or PhD)	0.062	0.115	0.512
Work setting			
Independent pharmacy (*Reference*)	-	-	-
Chain pharmacy	0.158	0.086	0.060
Attitudes score	0.430	0.080	0.000*
Barrier A			
Lack of perceived benefits of PBR	-0.256	0.066	0.002*
Barrier B			
Lack of institutional support for PBR	-0.041	0.063	0.263
Barrier C			
Lack of self engagement	0.186	0.073	0.312

## Discussion

Pharmacists are cornerstones of the health care system. The integration of current best evidence in daily pharmacy practice has been reported among pharmacists in different practice settings. Among those settings, community pharmacists were reportedly the least likely to utilize the current best evidence in everyday pharmacy practice [23].

As evident from the utilization score, pharmacists in this study showed high utilization of the current best evidence (mean scores >3.5). These findings are higher than the reported moderate level of utilization of current best evidence among pharmacists [2, 4, 24]. A lower level of evidence utilization has also been reported in the literature [12]. Pharmacists in this study showed a positive attitude toward PBR (attitude mean score >3.5). This finding is consistent with the literature where pharmacists have been shown to have a positive attitude towards PBR; most studies highlighted that there is a high level of interest and positive attitude towards PBR among pharmacists [15, 25].

This is the first study to explore the impact of pharmacists’ attitude towards PBR on their utilization of current best evidence in pharmacy practice; attitude toward PBR was a statistically significant predictor of pharmacists’ utilization of current best evidence; a positive attitude was associated with high utilization of current best evidence in daily practice (p<0.05). Despite the lack of studies on the impact of attitude of pharmacists attitude on their utilization of research in their practice, attitudes toward research in general, have been shown to relate to research utilization [26].

Although PBR is now more acknowledged and supported by pharmacy regulatory bodies worldwide, pharmacists have been reported to be reluctant to participate in PBR [27]. To address this reluctance in conducting PBR, we need to investigate factors that might hinder or encourage the involvement of pharmacists in PBR [25]. High workload, lack of workplace research culture, financial issues, lack of time, administrative support, research skills, and knowledge have all been reported as barriers to PBR [12, 13, 15]. Certain patterns of enthusiasm and contentment and other individual’s traits for contribution in PBR among pharmacists have also been reported as contributing factors for PBR [16]. Several studies reported barriers to utilization of current best evidence in daily pharmacy practice, including high clinical workload, and lack of basic skills and knowledge of critical appraisal [4, 6, 27]. Involving community pharmacists in PBR can potentially help break barriers to the utilization of the current best evidence in everyday pharmacy practice [3, 10, 25].

Lack of perceived benefit of BPR has been reported as one of the barriers to PBR as well as to the utilization of current best evidence [16].

In this study, we investigated several barriers to PBR those included; the lack of perceived benefits, lack of institutional support, and lack of self-engagements of community pharmacists to PBR. Also, there was a significant correlation between the lack of perceived benefit of PBR and their utilization of current best evidence (*p*<0.05). The lack of perceived benefit was found to be negatively associated with pharmacists’ utilization of the current best evidence.

Tackling these factors can result in increasing the involvement of pharmacists in PBR, and improving pharmacy practice. Being part of PBR can improve pharmacists’ knowledge and help in building research skills among pharmacists [10]. Research capacity building through PBR can have the potential to increase the utilization of the current best evidence in daily pharmacy practice among pharmacists. Pharmacists who participate in PBR are generally more skilled in extracting evidence from the literature and more likely to translate research evidence into pharmacy practice [12, 13, 25, 28]. It has been reported that pharmacists who tend to perform literature searches are more likely to intervene and suggest changes in drug therapy whenever needed, which can potentially improve health outcomes [13]. Several approaches have been proposed to enhance the translation of current best evidence into pharmacy practice among community pharmacists, which include continuing professional development and educational outreach visits [29]. Neither of these has proved to be effective in promoting more evidence utilization in a community pharmacy setting [29]. The effect of promoting PBR among community pharmacists has not been studied; this can be a promising mean of improving community pharmacists’ utilization and implementation of the most recent guidelines in their daily pharmacy practice.

## Limitation

Although the random selection of participants in this study helped in having unbiased representation of the larger population, a larger sample size would have helped to withdraw a more robust conclusion.

## Conclusion

In this study, pharmacists’ utilization of current best evidence was significantly impacted by their attitude toward PBR. The current study findings highlight the importance of integrating, supporting, and facilitating PBR in the community pharmacy setting in order to enhance the utilization of the current best evidence in everyday pharmacy practice.

## Supporting information

S1 Table Intercorrelations for pharmacists’ utilization of best current scientific evidence and predictor variables (N = 169). (DOCX)

S2 Table Results of exploratory factor analysis. (DOCX)

S1 File Questionnaire practice-based research. (PDF)
