# Supplementary material for: Community pharmacists’ attitudes toward practice-based research and their perceived utilization of scientific evidence
Source: PLoS One. 2022 Mar 15;17(3):e0264193. doi: 10.1371/journal.pone.0264193 (PMC8923436; doi:10.1371/journal.pone.0264193)
Supplement: S1 Table — (DOCX) [file pone.0264193.s001.docx]

| **S1 Table. Intercorrelations for Pharmacists’ Utilization of best current scientific evidence and Predictor Variables (N = 169).** | | | | | | | | | | |
| --- | --- | --- | --- | --- | --- | --- | --- | --- | --- | --- |
| Variables | Utilization | Gender | Age Group | Experience (years) | Levels of education/degree | Work setting | Attitudes | Lack of perceived benefits of PBR | Lack of institutional support for PBR | Lack of self engagement |
| Utilization | 1 | 0.001 | 0.019 | -0.109 | -0.013 | -0.053 | 0.477^*^ | -0.351^*^ | 0.075 | 0.068 |
| Gender |  | 1 | -0.08 | 0.067 | 0.061 | 0.206^*^ | -0.028 | -0.027 | 0.018 | -0.099 |
| Age Group |  |  | 1 | 0.352* | 0.128 | 0.032 | 0.027 | 0.15^*^ | 0.15^*^ | 0.1 |
| Experience (years) |  |  |  | 1 | 0.084 | 0.082 | 0.074 | 0.123 | 0.048 | 0.091 |
| Levels of education/degree |  |  |  |  | 1 | -0.076 | -0.001 | 0.078 | 0.137 | 0.228^*^ |
| Work setting |  |  |  |  |  | 1 | -0.189^*^ | 0.19^*^ | 0.153^*^ | -0.182^*^ |
| Attitudes |  |  |  |  |  |  | 1 | -0.237^*^ | 0.126 | -0.005 |
| Lack of perceived benefits of PBR |  |  |  |  |  |  |  | 1 | -0.051 | 0.093 |
| Lack of institutional support for PBR |  |  |  |  |  |  |  |  | 1 | 0.071 |
| **P*-value < 0.05 | | | | | | | | | | |
