# Supplementary material for: Community pharmacists’ attitudes toward practice-based research and their perceived utilization of scientific evidence
Source: PLoS One. 2022 Mar 15;17(3):e0264193. doi: 10.1371/journal.pone.0264193 (PMC8923436; doi:10.1371/journal.pone.0264193)
Supplement: S2 Table — (DOCX) [file pone.0264193.s002.docx]

| **S2 Table. Results of Exploratory Factor Analysis** | | | |
| --- | --- | --- | --- |
| **Statements** | **Factors** | | |
|  | **1** | **2** | **3** |
| The research is not reported in a clear and readable manner | 0.479 |  |  |
| The conclusions drawn from the research are not justiﬁed | 0.547 |  |  |
| I feel the beneﬁts of changing practice will be minimal | 0.685 |  |  |
| I am unaware of the research | 0.541 |  |  |
| I see few beneﬁts for myself | 0.614 |  |  |
| I do not see the value of research for practice | 0.563 |  |  |
| Lack of requisite skills to get involved in pharmacy practice research |  | 0.445 |  |
| Inability to provide the necessary financial commitment for pharmacy practice-based research |  | 0.495 |  |
| Time constraints in combining professional service with research |  | 0.551 |  |
| Additional staff required for conduct of pharmacy practice-based research is not |  | 0.552 |  |
| Lack of access to patient’s data hinder pharmacy practice-based research |  | 0.53 |  |
| Acquisition of additional training in research is essential for effective pharmacy practice research |  | 0.435 |  |
| Research reports/articles are not readily available (Literature access). |  |  | 0.523 |
| Statistical analyses are not understandable (Understandable statistics) |  |  | 0.594 |
| Implications for practice are not made clear |  |  | 0.654 |
| I don’t have time to read research articles |  |  | 0.548 |
| Physicians will not cooperate with implementation |  |  | 0.619 |
| Extraction Method: Principal Axis Factoring; Rotation Method: Varimax with Kaiser Normalization. | | | |
