# Supplementary material for: Community pharmacists’ attitudes toward practice-based research and their perceived utilization of scientific evidence
Source: PLoS One. 2022 Mar 15;17(3):e0264193. doi: 10.1371/journal.pone.0264193 (PMC8923436; doi:10.1371/journal.pone.0264193)
Supplement: S1 File — (PDF) [file pone.0264193.s003.pdf]

**Part 1: Socio-demographic characteristics**

1. Age (years) ______
2. Gender ☐ Male ☐ Female
3. Levels of education/degree:

   ☐ Pharmacist

   ☐ Doctor of pharmacy

   ☐ Master degree in pharmacy

   ☐ PhD in pharmacy
4. Time since working as a community pharmacist (years of experience):

   ☐ <5 years ☐ >=5years
5. Work setting:

   ☐ Independent pharmacy ☐ Chain Pharmacy
6. Research interest:

   ☐ Pharmacy Administration ☐ Basic Science (Pharmacogenomics, New structural drugs)

   ☐ Pharmacoeconomics/Epidemiology

   ☐ Pharmacy practice ☐ Hospital Pharmacy ☐ Therapeutics

   ☐ Pharmacokinetics ☐ pharmaceutics ☐ Others

**Part 2: Attitude towards practice-based research**					
**Statements**	**Strongly disagree**	**Disagree**	**Neutral**	**Agree**	**Strongly agree**
1. I like to read research studies related to pharmacy practice					
2. I shall be glad to be a part of research projects related to pharmacy practice					
3. I have faith in my capabilities to apprehend research and related terminologies concerned with pharmacy practice					

4. I am confident about my skills for designing research project related to pharmacy practice					
5. I am self-reliant in my skill for evaluating research terms of their application to pharmacy practice					
6. Pharmacy practice research is significant in recognizing and examining complications in pharmacy					
7. Pharmacy practice research is vital in pharmacy decision-making					

**Part 3: Utilization of best current evidence in pharmacy practices**					
**Statements**	**Strongly disagree**	**Disagree**	**Neutral**	**Agree**	**Strongly disagree**
Using best current evidence in daily practice					
Using databases or search engines to search journal articles					
Ability to identify clinical problems by using research and journal clubs					
Establish current best practices					
Being up to date with research to improve drug selection					
Reviewing research to help pharmacist with drug monitoring in daily practice					

**Part 4: Barrier and challenges to pharmacy practice-based research**					
**Barrier**	**Strongly disagree**	**Disagree**	**Neutral**	**Agree**	**Strongly agree**
1. Lack of requisite skills to get involved in pharmacy practice research					
2. Inability to provide the necessary financial commitment for pharmacy practice-based research					
3. Time constraints in combining professional service with research					
4. Additional personnel are to perform pharmacy practice-based research					
5. The lay-out of practice setting do not encourage research					

6. Lack of access to patient's data hinder pharmacy practice-based research					
7. Acquisition of additional training in research is essential for effective pharmacy practice research					
8. I feel I don't have enough authority to change patient care procedures (Insufficient authority)					
9. Research reports/articles are not readily available (Literature access).					
10. Statistical analyses can be difficult to understand					
11. There is insufficient time on the job to implement new ideas (Insufficient time)					
12. I feel the results are not generalizable to my own setting					
13. The facilities are inadequate for implementation					
14. Implications for practice are not made clear					
15. I don't have time to read research articles					
16. I feel incapable of evaluating the quality of the research					
17. Physicians will not cooperate with implementation					
18. The amount of research information is overwhelming					
19. The relevant literature is not compiled in one place					
20. The research has methodological inadequacies					
21. The literature reports conflicting results					
22. The research has not been replicated					
23. Other staffs are not supportive of implementation					
24. Research reports/articles are not published fast enough					
25. Administration will not allow implementation					
26. I am uncertain whether to believe the results of the research					
27. The research is not reported in a clear and readable manner					
28. The conclusions drawn from the research are not justified					
29. I feel isolated from knowledgeable colleagues with whom to discuss the research					
30. I feel the benefits of changing practice will be minimal					
31. I am unaware of the research					

32. There is not a documented need to change practice					
33. The research is not relevant to pharmacy practice					
34. I see few benefits for myself					
35. I do not see the value of research for practice					
36. I am unwilling to change/try new ideas					

Thank you for your participation in this anonymous survey
